# Lung Radiology and Pulmonary Function of Children Chronically Exposed to Air Pollution

**DOI:** 10.1289/ehp.8377

**Published:** 2006-04-20

**Authors:** Lilian Calderón-Garcidueñas, Antonieta Mora-Tiscareño, Lynn A. Fordham, Charles J. Chung, Gildardo Valencia-Salazar, Silvia Flores-Gómez, Anna C. Solt, Alberto Gomez-del Campo, Ricardo Jardón-Torres, Carlos Henríquez-Roldán, Milan J. Hazucha, William Reed

**Affiliations:** 1 Instituto Nacional de Pediatría, Mexico City, Mexico; 2 College of Health Professions and Biomedical Sciences, University of Montana, Missoula, Montana, USA; 3 Pediatric Imaging Section, Department of Radiology, University of North Carolina–Chapel Hill, Chapel Hill, North Carolina, USA; 4 Western New York Radiology, Buffalo General Hospital, Buffalo, New York, USA; 5 Pediatric Private Practice, Mexico City, Mexico; 6 Harvard South Shore Psychiatry Program, Brockton, Massachusetts, USA; 7 Departamento de Radiología e Imagén, Hospital Central Militar, Mexico City, Mexico; 8 Centro de Ciencias de la Atmósfera, Universidad Nacional Autónoma de México, Mexico City, Mexico; 9 Departamento de Estadística, Universidad de Valparaíso, Chile; 10 Center for Environmental Medicine, Asthma, and Lung Biology, University of North Carolina–Chapel Hill, Chapel Hill, North Carolina, USA

**Keywords:** air pollutants, chest X rays, children, high-resolution CT, hyperinflation, Mexico, ozone, particulate matter, small-airway disease, spirometry

## Abstract

We analyzed the chest radiographs (CXRs) of 249 clinically healthy children, 230 from southwest Mexico City and 19 from Tlaxcala. In contrast to children from Tlaxcala, children from southwest Mexico City were chronically exposed to ozone levels exceeding the U.S. National Ambient Air Quality Standards for an average of 4.7 hr/day and to concentrations of particulate matter (PM) with aerodynamic diameters ≤2.5 μm (PM_2.5_) above the annual standard. CXRs of Mexico City children demonstrated bilateral hyperinflation (151 of 230) and increased linear markings (121 of 230). Hyperinflation and interstitial markings were significantly more common in Mexico City children (*p* < 0.0002 and 0.00006 respectively). Mexico City boys had a higher probability of developing interstitial markings with age (*p* = 0.004). Computed tomography (CT) scans were obtained in 25 selected Mexico City children with abnormal CXRs. Mild bronchial wall thickening was seen in 10 of 25, prominent central airways in 4 of 25, air trapping in 8 of 21, and pulmonary nodules in 2 of 21. Only 7.8% of Mexico City children had abnormal lung function tests based on predicted values. These findings are consistent with bronchiolar, peribronchiolar, and/or alveolar duct inflammation, possibly caused by ozone, PM, and lipopolysaccharide exposure. The epidemiologic implications of these findings are important for children residing in polluted environments, because bronchiolar disease could lead to chronic pulmonary disease later in life.

Exposure to air pollutants produces adverse effects on lung development in children, leading to significant pulmonary function deficits as children reach adulthood (Gauderman et al. 2004). Epidemiologic studies of children strongly suggest that increased respiratory morbidity and mortality are related to chronic exposure to photochemical pollutants and particulate matter (PM) (Avol et al. 2001; Dockery et al. 1989; Gent et al. 2003; Gold et al. 1999; Kunzli et al. 2003; O’Neill et al. 2004; Pope and Dockery 1992). Residents of southwest Mexico City (SWMC) are exposed to daily ozone concentrations well above the National Ambient Air Quality Standards (NAAQS) established by the U.S. Environmental Protection Agency (Baez et al. 1995; Blake and Rowland 1995; Bonner et al. 1998; Bravo and Torres 2002). Currently, SWMC is classified as a serious nonattainment area (O_3_ range, 0.160–0.180 ppm) (U.S. Environmental Protection Agency 1992). PM levels are also above the NAAQS, with a yearly average of 78 μg/m^3^ for PM with aerodynamic diameters ≤10 μm (PM_10_) and 22 μg/m^3^ for PM with aerodynamic diameters ≤2.5 μm (PM_2.5_) (Alfaro-Moreno et al. 2002; Bravo and Torres 2002; Osornio-Vargas et al. 2003). Significant concentrations of lipopolysaccharides (LPS), aldehydes, volatile organic compounds (VOCs), nonmethane organic compounds, combustion-related metals, and alkaline hydrocarbons are repeatedly measured in the air (Baez et al. 1995; Blake and Rowland 1995; Bonner et al. 1998; Bravo and Torres 2002). Exposure to such a contaminated environment may pose a significant health risk for children. Because of the mild climatic conditions, children in Mexico City engage in play and outdoor physical activities throughout the year in late morning and afternoon when the diurnal pollutant levels are at their maximum (Villarreal-Calderon et al. 2002). Schoolchildren in Mexico City are reported to have an association between daily ambient O_3_ concentrations and acute decrements in forced expiratory volume in 1 sec (FEV_1_) (Castillejos et al. 1995), as well as decrements in peak expiratory flow (PEF) associated with O_3_ and PM exposures in the preceding 1–2 weeks (Gold et al. 1999). Our laboratory reported a significant seasonal drop in forced vital capacity (FVC) and FEV_1_ associated with a 6-month period of high O_3_ and PM_10_ (Calderón-Garcidueñas et al. 2003). Thus, children in SWMC present spirometric changes that can be related to chronic exposures to their polluted environment.

A pilot study evaluating chest X rays (CXRs) in clinically healthy children from a low-polluted area versus children in SWMC showed bilateral symmetric hyperinflation in 50% of SWMC children and in 5% of controls, suggesting that hyperinflation may be associated with chronic exposures to air pollutants (Calderón-Garcidueñas et al. 2000). In this report we present data from a new cohort of SWMC and control children. The primary purpose of this study was *a*) to score plain CXRs of clinically healthy children, permanent residents of either SWMC or the control city, for hyperinflation and linear markings, two components of the Brasfield score system that is used for cystic fibrosis radiologic evaluation (Brasfield et al. 1980); *b*) to analyze high-resolution lung computed tomography (CT) scans of the lungs of selected Mexico City children with abnormal CXRs to confirm the abnormal CXR findings; and *c*) to analyze the relationship between the spirometric and the radiologic findings.

Hyperinflation and linear markings are radiologic evidence of bronchiolar, peribronchiolar, and/or alveolar duct inflammation (Calderón-Garcidueñas et al. 2003). Pulmonary radiographs are essential adjuncts to the evaluation and diagnosis of suspected pulmonary disease, and abnormal findings should be interpreted in the context of the clinical and environmental settings. In this study, CXR abnormalities were seen in clinically healthy children living in an urban environment with significant concentrations of O_3_ and PM who have no risk factors for lung diseases.

## Materials and Methods

### Study areas

The two areas selected for this study were SWMC and Tlaxcala, representative of high- and low-polluted urban areas, respectively. Mexico City is located in a high mountain basin 2,250 m above sea level. SWMC was selected based on the significant concentrations of O_3_, PM_10_, and PM_2.5_ recorded all year long (Blake and Rowland 1995; Bonner et al. 1998; Bravo and Torres 2002). Tlaxcala is located 130 km east of Mexico City at 2,252 m above sea level. Although ambient air pollution levels are not monitored year-round in Tlaxcala, the available data for years 1994–2000 suggest that the levels of criteria air pollutants do not regularly exceed their respective U.S. air quality standard (Torres-Jardón R, personal communication). Thus, the two cohorts in this study were chronically exposed to significantly different levels of outdoor air pollution for at least 7 years before the collection of radiologic and spirometric data.

### Subject recruitment

This study complied with all applicable requirements and regulations concerning the protection of the rights of human subjects. The study protocol was approved by the Review Board for Human Studies Committee at the Instituto Nacional de Pediatría, Mexico City. Written informed consent was obtained from parents, and oral consent from children, before participation in the study. All children were Mexican. Mexico City children were recruited from two sources: a school located < 6 miles from the two selected pollutant monitoring stations, and children of staff at the Instituto Nacional de Pediatría. Control children from Tlaxcala were also recruited from a single school and from children of the staff at the selected school.

Recruitment was done in the summer of 1999 and 2000. Participation was limited to children who fulfilled the following criteria: lifelong residence in SWMC or in the control city; nonsmoking households and negative personal smoking history and environmental tobacco exposure, including exposure to maternal smoking *in utero*; both residence and school ≤5 miles from the fixed pollutant monitoring station; no known exposures to local sources of pollutants; unremarkable clinical histories, including negative history of premature birth, bronchitis, pneumonia, asthma or asthma-like symptoms, respiratory infections, and no hospitalizations; ages ranging from 5 to 13 years; negative family history of atopic diseases; and no indoor pets.

Recruitment was done by word of mouth. The parents of the participating children had another child or a relative participating in previous clinical studies by the researchers. The recruitment sequence included an initial parent interview, where the nature of the study and the inclusion criteria were explained to the interested parents, followed by an interview with the child and a consent form signature. Subsequent visits included the physical examination with the pediatrician, the spirometric studies, and the CXR. The high-resolution CTs were scheduled only after selection of children with abnormal CXRs by the two radiologist groups in Mexico and the United States. The average period between the CXR and the computed tomography (CT) was 9.2 ± 2 months because of the requirement of an additional consent form for the CTs. Participating children were well nourished, slept in bedrooms without carpeting, and had open windows for ventilation. All households had kitchens separated from the living and sleeping areas and used gas for cooking.

### CXR and CT scans

Frontal and lateral CXRs were obtained from each control and exposed child. Three pediatric board-certified radiologists interpreted the radiographs. The films were scored, and the readers were blinded to the child’s city of residence. The films were analyzed using two components of the Brasfield score: hyperinflation and linear markings (Brasfield et al. 1980). The films were scored on a 4-point scale with 0 indicating no radiographic finding and 1, 2, or 3 indicating mild, moderate, or severe presence of each radiographic finding. After scoring the CXRs, 25 children with mild to severe hyper-inflation were selected to have CT scans. Scans were performed at a peak kilovoltage of 120 kVp and 80 mA with a 1.2-sec scan time. Slice thickness varied from 2 to 5 mm. CT scanning was performed in full inspiration and expiration in 21 children. Three additional children had only inspiratory CTs, and one only an expiratory CT. Scans were evaluated for presence or absence of air trapping, ground glass opacities, bronchiectasis, and bronchial wall thickening. Pediatric radiologists participating in this study had previously read CXRs from Mexico City children mixed with films of North Carolina healthy children and North Carolina cystic fibrosis patients in order to assure accuracy in their evaluations and establish interobserver variations in the calculations of the percentage of observed agreements and had obtained an average weighted kappa statistic of 0.76 (Calderón-Garcidueñas et al. 2000). For this set of radiologic studies, the radiographs were scored independently by the Mexico City radiologist and by consensus of the two North Carolina radiologists. The final score resulted from an evaluation of the two readings. In three Mexico City cases, the final score was based on the North Carolina reading score; in the remaining, the readings were identical for the three radiologists.

### Spirometry

Pulmonary function tests (PFTs) were always performed weekdays between 0830 hr and 1100 hr according to the European Working Party Standardization of Lung Function Tests protocol (Quanjer et al. 1993) and the American Thoracic Society (1991) guidelines. A rolling-seal spirometer (S&M Instruments Ltd., Doylestown, PA, USA) coupled with computerized data acquisition software was used. Because the lungs’ volumes are essentially anatomic compartments within the human chest, their values are expressed as body temperature, ambient pressure, and water vapor saturation conditions. The following PFT variables were included in the data set: FVC, FEV_1_, PEF, average forced expiratory flow (FEF) over the middle 50% of the FVC flow rate (FEF_25–75_), and FEF at 75% of expired volume (FEF_75_). All the PFT variables are expressed as percentage of predicted values.

### Pollutant monitoring stations

Atmospheric pollutants were monitored at two monitoring stations, Pedregal and Coyoacan, located in SWMC downwind of the major diurnal emissions in metropolitan Mexico City. Pedregal station measured average hourly levels of O_3_ and PM_10_. PM_2.5_ was monitored at the Coyoacan station. For O_3_ and PM_10_ exposure, we examined three measures: the maximal concentrations, the number of hours equal to or above the NAAQS, and the time of occurrence of peaks. Periodic air pollutant monitoring data from Tlaxcala for 1994–2000 showed that levels of criteria air pollutants did not exceed their respective U.S. NAAQS.

### Statistical analysis

We determined the significance of the differences in the 2 × 4 contingency tables between hyperinflation severity and cohort and interstitial markings severity and cohort using an exact Bayes test for independence in rank × category contingency tables. We calculated bivariate correlations using Spearman’s rank correlation test. A model of predicted probabilities of developing interstitial markings was developed taking into account age, sex, and the interaction between age and sex. All the statistical computations were performed with SAS software (version 8.2; SAS Institute Inc., Cary, NC, USA). We considered a two-sided type I error rate of 0.05 with a power of 0.90 to detect 5% of difference between the two cohorts (*p* < 0.05) to be significant. Aggregate results are given as mean ± SE.

## Results

### Air quality data

**Figure 1:**
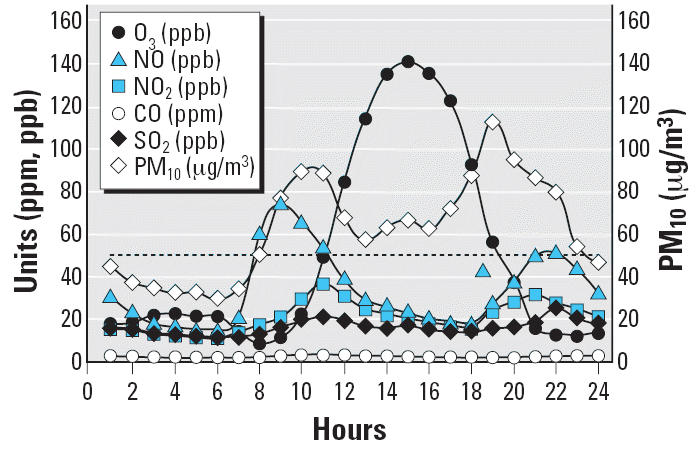
The typical 24-hr pattern of key air pollutants in southwest metropolitan Mexico City averaged over 31 days for the month of January 1999. Left scale: O_3_, nitric oxide, NO_2_, carbon monoxide, sulfur dioxide; right scale: PM_10_. The horizontal dashed line at 50 μg/m^3^ represents the current yearly PM_10_ standard. There is an average of 4 ± 1 hr/day with O_3_ values above 0.08 ppm. The average yearly PM_10_ level is 48 μg/m^3^, and that for PM_2.5_ is 21 μg/m^3^.

The 24-hr pattern of key air pollutants in SWMC averaged over 31 days for January 1999 is illustrated in Figure 1. For the study years 1999 and 2000, the SWMC showed an average of 4 ± 1 hr/day with O_3_ above 0.08 ppm, whereas the average annual PM_10_ values were 48 μg/m^3^ and 45 μg/m^3^, and PM_2.5_ values were 21 and 19 μg/m^3^, respectively. O_3_ and PM_2.5_ were consistently above the NAAQS. Children were exposed to significant amounts of air pollutants between 0800 hr and 1800 hr, coinciding with the hours of daylight outdoor activities.

### Demographics

All participating children were from middle-class families in both Mexico City and Tlaxcala. Parents were professionals, administrative personnel, and white-collar workers who lived in single-family houses. No occupational exposures to toxicants were reported by parents or close relatives. A total of 292 children were recruited for the study. Of that group, 271 were from SWMC and 21 from the control city. A total of 43 children were excluded from the study after initial enrollment. Two were excluded from the control group, one because of pigeons living on the roof and the other because of an upper respiratory tract infection at the time of CXR. Forty-one from the SWMC group were excluded for the following reasons: 13 for exposure to tobacco smoke, 11 for incomplete data, 6 for frequent weekend travel outside Mexico City, 5 for chronic sinusitis on physical exam, 4 for exposure to paint and paint solvents, 1 for exposure to airplane model glue, and 1 for exposure to an indoor bird.

The control children included 10 girls and 9 boys, 10.8 ± 0.6 years of age, with an average daily outdoor time of 4.4 ± 2.5 hr. The SWMC group included 108 girls and 122 boys, 9.78 ± 2.95 years of age, with an average outdoor time of 3.9 ± 2.3 hr/day.

### Radiologic findings

**Figure 2:**
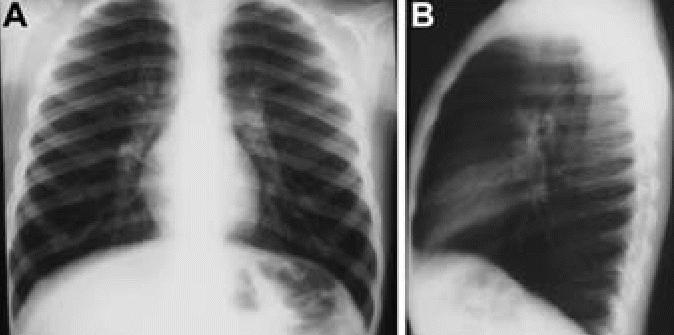
Eleven-year-old boy with frontal (*A*) and lateral (*B*) CXRs that demonstrate hyperinflation. The lateral film shows an increase in the anterior clear space, increased anterior–posterior diameter, and flattening of the hemidiaphragms.

**Figure 3:**
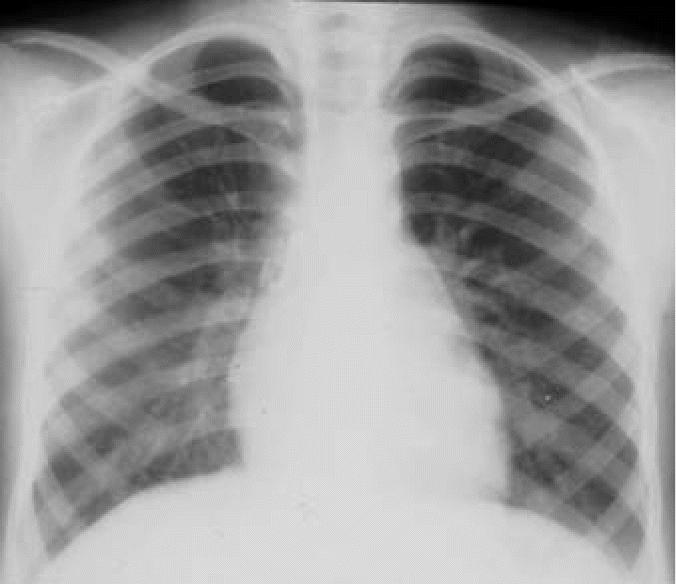
Ten-year-old boy with a frontal CXR that demonstrates subtle increased linear markings.

Hyperinflation and linear markings are not described in normal children. CXR demonstrated mild bilateral hyperinflation in one child from the control city (5.3%). In SWMC children, mild to severe hyperinflation was present in 151 of 230 (65.6%). In addition, mild or moderate linear markings were observed in 121 of 230 (52.6%) SWMC children, whereas linear markings were not seen in control children. Representative CXR showing hyperinflation and linear markings in SWMC children are shown in Figures 2 and 3, respectively, and the range of scores for hyperinflation and linear markings is shown in Table 1. The frequencies of both hyperinflation and interstitial markings were significantly higher in Mexico City children (*p* < 0.0002 and 0.00006, respectively), indicating an association of hyperinflation and interstitial markings with residence in Mexico City.

Table 2 shows the calculated probabilities of SWMC boys and girls for developing interstitial markings based on age, sex, and the combination of both. Boys had a higher predicted probability of developing interstitial markings with age (*p* = 0.004)(Table 2), whereas the risk in girls decreased with age. In both cohorts, boys had on average 1.2 hr more outdoor exposure than did girls.

**Figure 4:**
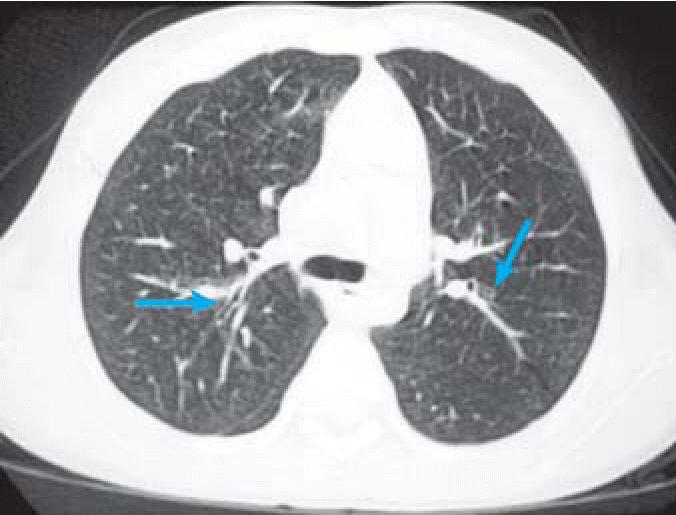
Inspiratory chest CT demonstrating mild peribronchial thickening (left arrow) and minimal airway dilatation (right arrow) in an 11-year-old boy.

**Figure 5:**
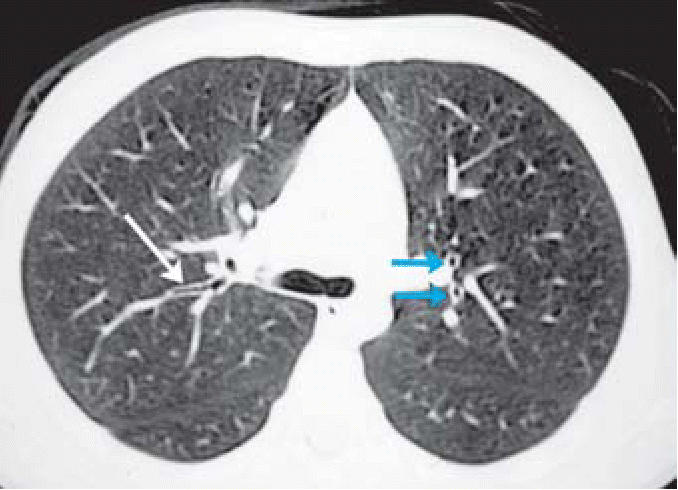
High-resolution axial CT of a 10-year-old boy demonstrating mildly dilated central airways (blue arrows) and mild peribronchial thickening (white arrow).

**Figure 6:**
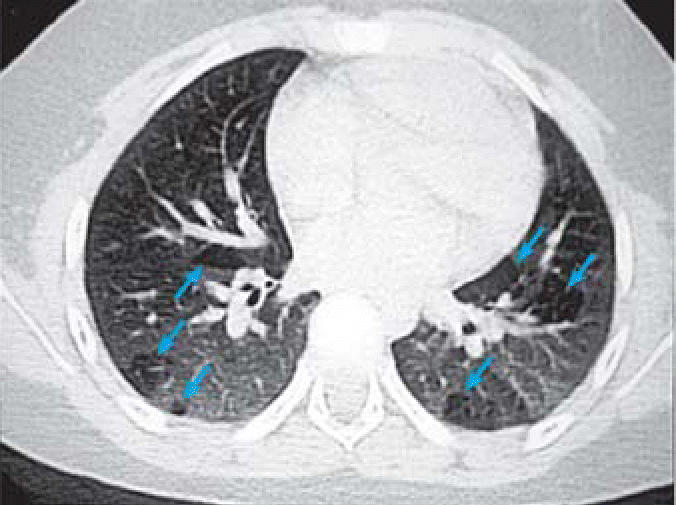
High-resolution expiratory CT of a 9-year-old boy demonstrating air trapping at the level of the secondary pulmonary lobule (arrows).

**Figure 7:**
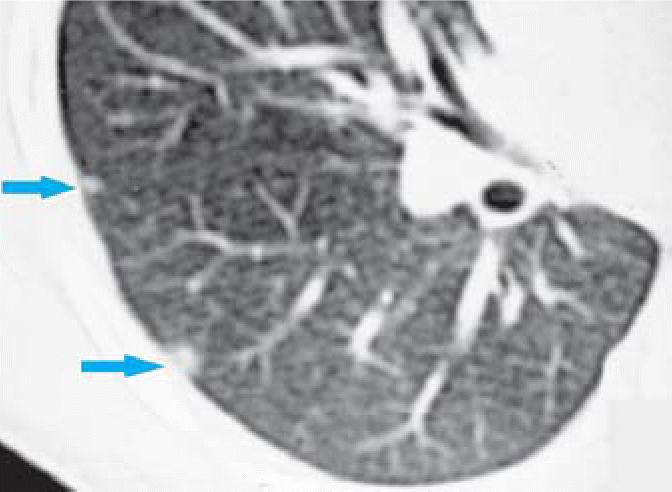
High-resolution CT of a 12-year-old demonstrating subpleural pulmonary nodules (arrows).

CT scans were performed on a subset of children with abnormal CXR. Mild bronchial wall thickening was seen in 14 of 25 CTs (eight males, six females; Table 3, Figure 4); 4 of 25 (one male, three females; Table 3) showed prominent bronchi (Figure 5), and 8 of 21 (four males, four females; Table 3) (the cases performed in full inspiration and expiration) showed air trapping at the level of the secondary pulmonary lobule (Figure 6). Two children exhibited peripheral nodules not seen on CXR (Figure 7). Neither ground glass opacities nor septal thickening was seen. The CTs of 10 of 25 children (five males, five females; Table 3) with abnormal CXRs were read as normal.

### Spirometry

We measured pulmonary function in 77 of 230 SWMC children and 19 of 19 controls. Six SWMC children (three males, three females) (7.8%) had low FEV_1_ defined as the ratio of observed to expected FEV_1_ < 80% (Table 4). In addition there were significant deficits in FVC and FEF_25–75_ for most of these children. One child had a normal CXR, whereas the CXR of the other five showed hyperinflation or interstitial markings or both. Children with CXR hyperinflation who had PFTs (*n* = 55) showed a significant association between hyperinflation and percent predicted FVC (*r* = 0.49; *p* = 0.0003), and percent predicted FEV_1_ (*r* = 0.50; *p* = 0.0003). Control children had percent predicted FVC and percent predicted FEV_1_ within the predicted range.

## Discussion

The two areas selected for this study were Tlaxcala and SWMC, representative of low- and high-polluted urban areas, respectively. Ambient air pollution levels are not monitored year-round in Tlaxcala, but periodic monitoring indicated that the levels of criteria air pollutants did not regularly exceed their respective U.S. NAAQS for the 7 years preceding this study (Torres-Jardón R, personal communication). In contrast, during the same period, criteria air pollutant levels in Mexico City regularly exceeded their respective NAAQS, especially O_3_ and PM. The annual emissions of pollutants in Mexico City is > 16 million tons, of which 65% come from vehicles and the rest from industry (Bravo and Torres 2002). Vehicle emissions account for much of the carbon monoxide, nitrogen dioxide, and VOCs (Baez et al. 1995; Blake and Rowland 1995; Bonner et al. 1998; Bravo and Torres 2002). Significant concentrations of PM, O_3_, aldehydes, VOCs, nonmethane organic compounds, alkaline hydrocarbons, and LPS are repeatedly measured in Mexico City air (Baez et al. 1995; Blake and Rowland 1995; Bonner et al. 1998; Bravo and Torres 2002).

Previous comparative studies have demonstrated nasal, lung, and cardiac pathology in healthy SWMC mongrel dogs, whereas dogs from Tlaxcala exhibited minimal pathology (Calderón-Garcidueñas et al. 2001a, 2001b). Clinical studies of SWMC children have shown nasal pathology, decrements in respiratory function, and an imbalance of systemic pro- and anti-inflammatory cytokines (Calderón-Garcidueñas et al. 2001c, 2003). In this study, clinically healthy children with no risk factors for lung diseases but who were exposed to a polluted urban atmosphere with O_3_ and PM as the major pollutants exhibited a significant increase in the frequency of hyperinflation and interstitial markings on CXRs. Hyperinflation is a physiologic consequence of bronchiolar disease (Franquet and Stern 1999; Greenough et al. 1991; Hogg 2004; Hogg et al. 2004), whereas linear markings most likely reflect a disease process with a bronchiolar, peribronchiolar, and/or alveolar duct inflammatory component (Eschenbacher et al. 1999; Grum and Lynch, III 1992; Papiris et al. 1999). The CXR findings in Mexico City children are consistent with lung pathology described in SWMC dogs (Calderón-Garcidueñas et al. 2001b). These dogs had significant airway pathology at the bronchiolar level, including epithelial and smooth muscle cell hyperplasia, peribronchiolar fibrosis, and extension of a chronic inflammatory peribronchiolar infiltrate into adjacent vascular structures (Calderón-Garcidueñas et al. 2001b). Chronic bronchiolitis, present in Mexico City dogs, is a common denominator in chronic bronchitis and emphysema and a factor contributing to airflow limitation in chronic obstructive pulmonary disease (COPD) (Hogg 2004; Hogg et al. 2004; Jeffery 1998). The risk for developing chronic lung disease later in life could be higher for boys, because they have an increased probability of developing interstitial markings, an observation explained at least partly by the fact that boys spend considerably more time outdoors than girls and engage in moderate to intense exercise (Villarreal-Calderon et al. 2002).

In this group of Mexican children, CT demonstrated peribronchial thickening, airway dilatation, and mild air trapping. CT also demonstrated subpleural nodules that were not visualized on the plain films. CT scans of the lungs of healthy dogs from SWMC have also demonstrated pulmonary nodules (Mora-Tiscareño A, unpublished observations). In these dogs, the nodules were found to contain densely packed macrophages loaded with PM (Calderón-Garcidueñas et al. 2001b). Healthy nonsmoking Mexico City teenagers who die suddenly (accidental fatalities) also show sub-pleural accumulation of macrophages loaded with PM (Calderón-Garcidueñas L, unpublished data). Thus, the subpleural nodules in these Mexico City children may have similar pathology.

The pathology observed in Mexico City children and dogs is likely related to exposure to O_3_ and PM, which are known to target respiratory bronchioles (Camner et al. 1997; Eustis et al. 1981; Harkema et al. 1993; Howard-Reed et al. 2000; Kreyling et al. 1999; Vernooy et al. 2002). O_3_ and PM_10_ concentrations are above the NAAQS in SWMC, and although annual PM_2.5_ concentrations fluctuate slightly above and below the current standard, there may be synergistic effects of exposure to the complex mixture of pollutants in Mexico City air. Adamson et al. (1999) demonstrated that inhalation of urban dust at concentrations that cause few lung effects when inhaled alone can potentiate O_3_ toxicity, particularly in the vicinity of the alveolar duct where interstitial inflammatory cells accumulate, the anatomical target of air pollutants in both children and dogs residing in Mexico City (Calderón-Garcidueñas et al. 2001b; Calderón-Garcidueñas L, unpublished data).

Oxides of nitrogen (NO_x_) are another criteria air pollutant present in Mexico City air that can cause respiratory tract inflammation. Like O_3_ and PM, NO_2_ injures the respiratory tract and has its greatest effect upon respiratory bronchioles (Chauhan et al. 1998). However, the concentration of NO_2_ in Mexico City air is substantially lower than that of O_3_ (Figure 1), and NO_2_ is less toxic than O_3_ at the same concentration. Another NO_x_, nitric oxide, is a potent oxidant that is more abundant than NO_2_ in Mexico City air (Figure 1) and that at high concentrations can cause pulmonary injury and inflammation. Thus, it is possible that NO_x_ may interact with O_3_ and PM to exacerbate their effects on terminal bronchioles.

The LPS content in Mexico City PM_10_ samples ranges from 15.3 to 20.6 ng/mg (Bonner et al. 1998). LPS is a potent inducer of inflammation. Jansson et al. (2004) reported that intratracheal instillation of LPS in rats produces lung edema; acute inflammation with increased bronchoalveolar lavage concentrations of tumor necrosis factor-α, interleukin-1β, and monocyte chemoattractant protein-1 (MCP-1); and lung hyperinflation as determined by increased excised lung gas volume. Thus, LPS could be playing a role in the pathogenesis of the hyperinflation we observed in Mexico City children.

Some metals present in Mexico City PM are potentially capable of inducing inflammation. The most abundant metals encountered in Mexico City PM are calcium, iron, potassium, and zinc. In the fine fraction (PM_2.5_) there is a clear grouping of potassium, titanium, iron, calcium, and silicon, which are typically associated with PM originating from soil (Alfaro-Moreno et al. 2002). Metals typically present in fuel oil include chromium, nickel, and vanadium. High correlations are found between zinc, copper, and manganese in Mexico City PM, elements associated with industry or traffic (Alfaro-Moreno et al. 2002). The traffic contribution is present only in the fine PM fraction (Alfaro-Moreno et al. 2002).

Small-airway diseases in children present a diagnostic challenge because the clinical presentation and the radiographic features are nonspecific (Copley and Bush 2000; Koh and Hansell 2000; Kuhn and Brody 2002). Further, small-airway disease–associated shortness of breath and airflow limitation are patent only late in the pathogenesis of chronic pulmonary disease (Jeffery 1998), so it is imperative that tools capable of diagnosing early stages of chronic pulmonary disease are employed in environmental field studies. We show here that CT scans might serve as useful noninvasive means to further evaluate children who have an abnormal CXRs, to confirm the CXR findings, and to uncover lesions not seen in plain CXRs. Important issues that restrict the use of CT in children are radiation dose, availability, and observer experience (Copley and Bush 2000). Children with significant CXR and CT findings may represent the children who will have the most pronounced response to environmental pollutants, and this information could be used to provide a focused intervention for those children most at risk.

Ten of the 25 CTs of children with a diagnosis of hyperinflation by CXR showed no abnormal findings. This discrepancy could be explained by the observation that hyperinflation is a dynamic condition (Palecek 2001) and therefore could change over weeks depending on the pollutant exposures. For this study, the time interval between the abnormal CXRs and the CTs was 9.2 ± 2 months. Under ideal circumstances, the CXR should be followed immediately by the CT.

The long-term consequences of chronic or intermittent hyperinflation in children are unknown. Chronic hyperinflation affects respiratory muscle interaction, increases the rib cage contribution to chest wall motion, and reduces the abdominal contribution (Decramer 1997). Further, hyperinflation causes a dropout of diaphragm sarcomeres and increases the recruitment of expiratory muscles (De Troyer 1997; Decramer 1997). Because the exposed children have lifetime residencies in Mexico City, the effects of pollutants upon their respiratory system are both early and sustained, and more important, they occur at a very critical period in lung development. The development of the distal bronchioles and alveoli occurs primarily between birth and 2 years, with significant growth continuing up to 8 years of age (Mercer et al. 1994; Stick 2000; Thurlbeck 1975). Consequently, any factors that alter airway growth during childhood are likely to affect adult lung function (Stick 2000). Thus, these children exposed to significant concentrations of air pollutants potentially have an altered alveolar development and suffer adverse effects on lung function growth, similar to that experienced by children in Southern California (Gauderman et al. 2004).

There was a striking difference in the frequencies of CXR abnormalities (65.5%) and the deficits in FEV_1_ (7.8%). We used the same criteria for clinically impaired lung function (FEV_1_ < 80% predicted) as Gauderman et al. (2004), and our proportion of children, of average age 9.78 ± 2.95 years, with low FEV_1_ was similar to what Gauderman et al. reported for 18-year-olds residing in the Mira Loma, Riverside, and Upland Southern California communities with the highest levels of PM_2.5_ and in the same annual range as the SWMC values. Given the marked difference in our Mexico City population between the proportion of children with CXR and PFT abnormalities, PFTs may considerably underestimate the adverse health effects of exposure to ambient air pollutants. Inflammation and structural abnormalities in the small airways are considered the most important contributors to the FEV_1_ deficit in conditions such as COPD, although airflow limitation is patent only late in the pathogenesis of chronic pulmonary disease (Jeffery 1998).

Pediatricians who see patients residing in polluted urban areas and children living close to busy highways or industrial complexes should consider exposure to air pollution as an etiology of hyperinflation and increased linear markings on CXRs. Pediatricians and radiologists should be familiar with the air pollutant patterns in their cities and be aware that both outdoor and indoor air pollution are equally important in terms of children’s health effects. CXR in seemingly normal children may reveal hyperinflation and increased linear markings. CT may have a role in further evaluating children with CXR abnormalities. Finally, children may benefit from indoor play during peak O_3_ and PM concentrations.

## Conclusions

We have found an association between chronic exposures to severe urban air pollution and a significant increase in abnormal CXRs and lung CTs, suggestive of a bronchiolar, peribronchiolar, and/or alveolar duct inflammatory process, in clinically healthy children with no risk factors for lung disease. Although the frequency of pulmonary function deficits were also increased, they were not as common as lung radiologic changes.

## Figures and Tables

**Table 1:** Range of scores for hyperinflation and interstitial markings in children from Tlaxcala and SWMC.

	Severity			
Reading/cohort (*n*)	0	+	++	+++
Hyperinflation				
Tlaxcala	18	1	0	0
SWMC*	79	72	56	23
Interstitial markings				
Tlaxcala	19	0	0	0
SWMC**	109	112	9	0

Hyperinflation was severe in 15% of children with hyperinflation by CXR.

* Significantly different from Tlaxcala cohort (*p* < 0.0015).

** Significantly different from Tlaxcala cohort (*p* < 0.00006).

**Table 2:** Calculated odds of SWMC boys versus SWMC girls developing interstitial markings based on age, sex, and the combination of both.

	*b*	*z*	*p* > |*z*|
Age	–0.14725	–2.279	0.023
Sex	–2.3777	–2.454	0.014
Age and sex	0.27763	2.874	0.004

Abbreviations: *b*, raw coefficient; *z*, *z*-score for test of *b* = 0; *p* > |*z*|, *p*-value for *z*-test. Probabilities for boys increased with age, likely the result of their significantly higher outdoor exposures and their moderate to severe physical exercise while outdoors.

**Table 3:** High-resolution lung CT findings in SWMC children.

				CXR	
Subject sex/age (years)	Bronchial wall thickening	Air trapping	Bronchial dilatation	HI	IM
M 6	1	2	0	3	0
M 6	1	0	0	3	1
M 7*a*	0	—	0	1	1
M 7*a*	0	—	0	3	2
M 8	1	1	0	1	2
M 9	1	1	0	3	1
M 9	1	0	0	2	0
M 10	0	0	0	1	0
M 10	0	0	0	2	0
M 10	1	2	1	1	1
M 11	1	0	0	1	1
M 12	1	0	0	3	1
M 15*a*	0	—	0	2	1
F 6	0	0	0	1	0
F 6	0	0	0	1	2
F 8	0	0	0	3	1
F 8	0	0	0	1	0
F 8	0	0	0	2	1
F 8*a*	1	—	0	1	1
F 8	1	1	0	2	0
F 9	1	2	1	2	1
F 10	0	1	0	2	1
F 11	1	0	1	3	1
F 12	1	0	1	1	1
F 12	1	1	0	1	1

Abbreviations: F, female; HI, hyperinflation; IM, interstitial markings; M, male. Twenty-five children with abnormal CXRs had lung CTs. Bronchial wall thickening was seen in 14 of 25, air trapping in 8 of 25, and bronchial dilatation in 4 of 25. Ten CTs were read as unremarkable several months after the CXR was abnormal. Ratings are 0–3, where 0 is absence of pathology and 3 is conspicuous pathology.

a Child with only inspiratory or expiratory CT (—).

**Table 4:** Subset of SWMC children with abnormal PFTs (FEV_1_ < 80% predicted).

Sex/age (years)	FVC	FEV_1_	FEF	FEF_25–75_	FEF_75_	HI	IM
M 6	97.2	76.2	41.1	47.6	43.5	0	0
M 10	48.3	52.7	42.5	67.9	74.8	1	0
M 11	44.3	50.7	51.6	97.4	127	3	0
F 8	69.4	75.2	55.5	57.8	95.4	2	2
F 8	77.7	59.4	52.2	29	23.2	0	2
F 9	64.1	71.1	57.4	88.4	105	2	0

Abbreviations: HI, hyperinflation by CXR; IM, interstitial markings by CXR. In this subset of children, three boys and three girls had abnormal PFTs (6 of 77). One child had a normal CXR, whereas the others had hyperinflation, interstitial markings, or both.
